# GPER, 8-isoprostaglandin, and tau protein as potential biomarkers in supraventricular tachycardia: An observational study

**DOI:** 10.1097/MD.0000000000049850

**Published:** 2026-07-17

**Authors:** Ünal Öztürk, Nuray Üremiş, Ergül Belge Kurutaş

**Affiliations:** a Department of Cardiology, Medical Faculty, Kahramanmaraş Sütçü İmam University, Kahramanmaraş, Turkey; b Department of Medical Biochemistry, Medical Faculty, Kahramanmaraş Sütçü İmam University, Kahramanmaraş, Turkey.

**Keywords:** arrhythmia, cardiac biomarkers, estrogen, neurodegeneration, oxidative stress, supraventricular tachycardia

## Abstract

Supraventricular tachycardia (SVT) is a common arrhythmic disorder characterized by abnormal electrical impulses arising from supraventricular tissues, most commonly the atria or atrioventricular node. This study aimed to investigate the association between G protein-coupled estrogen receptor (GPER) levels and biomarkers of oxidative stress and neurodegeneration in the pathophysiology of SVT. This study included 51 patients diagnosed with SVT and 30 healthy controls. Plasma levels of the oxidative stress marker 8-iso-prostaglandin F2α (8-iso-PGF2α), the neurodegeneration-associated protein Tau, and GPER were measured using ELISA kits. Correlations between GPER and the studied biomarkers were analyzed, and the diagnostic performance of each biomarker was evaluated using ROC analysis. Pro-BNP levels were higher in patients with SVT compared to the control group (*P* = .05). GPER, Tau, and 8-iso-PGF_2_α levels were significantly higher in the SVT group compared to the control group (*P* < .001 for all 3 biomarkers). ROC analysis demonstrated that 8-iso-PGF_2_α had the highest diagnostic accuracy, while Tau (AUC = 0.986) and GPER (AUC = 0.981) also showed strong discriminative performance. GPER, a well-established marker of estrogen-mediated cellular signaling, emerges as a significant biomarker in patients with SVT. These findings suggest that GPER-mediated signaling pathways may be associated with systemic oxidative damage and neuronal effects in the pathophysiology of SVT.

## 1. Introduction

Supraventricular tachycardia (SVT) is a type of arrhythmia characterized by sudden onset and rapid heart rhythm caused by abnormal electrical impulses originating above the ventricles, typically in the atria or atrioventricular node.^[1]^ The impairments in cardiac electromechanical function that emerge with aging are largely based on the weakening of cellular antioxidant defense mechanisms and the consequent increase in reactive oxygen species (ROS).^[2]^ Among the primary targets of ROS are voltage-dependent sodium (Na^+^), potassium (K^+^), and L-type calcium (Ca^2+^) channels.^[3]^ The functional modification of these ion channels affects the action potential duration, shortens refractory periods, and thereby creates a substrate for the development of tachycardic beats.^[4]^ The metabolic stress and increased ROS induced by rapid heart rates lead to peroxidative damage in myocardial membrane lipids.^[5]^ 8-iso-PGF_2_α, which is generated as a result of this damage, can be considered a trigger of potential arrhythmogenic mechanisms.

Although the tau protein is not directly expressed in cardiac tissue, it is indirectly associated with arrhythmia through autonomic dysfunction and its effects on neural cardiac regulation that develop as a result of neurodegenerative processes.^[6]^ In particular, tau hyperphosphorylation and aggregation may lead to a reduction in cardiovagal tone by disrupting the structural and functional integrity of the central autonomic network.^[7]^ This condition results in arrhythmogenic effects, such as a decrease in heart rate variability and impairment of cardiac electrical stability.

GPER activation in cardiac tissue plays a decisive role in intracellular calcium dynamics and ion channel functions.^[8]^ In the literature, PI3K/AKT, ERK1/2, and cAMP-PKA pathways activated via GPER have been shown to stabilize action potential duration and regulate repolarization in cardiac myocytes.^[9]^ In the literature, it has been shown that PI3K/AKT, ERK1/2, and cAMP-PKA pathways activated via GPER exert action potential–stabilizing and repolarization-regulating effects in cardiomyocytes.^[10,11]^ However, reductions in GPER expression or functional impairments are associated with an increased risk of arrhythmia, particularly during the postmenopausal period.^[12]^

This study aims to comprehensively evaluate the role of GPER in the molecular pathophysiology of supraventricular tachycardia in the context of 8-iso-PGF_2_α–mediated oxidative stress and Tau-associated neurodegenerative processes.

## 2. Materials and methods

### 2.1. Study population

This study was conducted in collaboration between the Emergency Department, Cardiology Outpatient Clinic, and Medical Biochemistry Department of Kahramanmaraş Sütçü İmam University (KSÜ) Research Hospital and included patients who presented with electrocardiographically confirmed active supraventricular tachycardia (SVT) prior to any pharmacologic or procedural intervention. The study was approved by the KSÜ Medical Research Ethics Committee under protocol number 2025/21-157. Inclusion criteria comprised electrocardiographic confirmation of active paroxysmal supraventricular tachycardia (PSVT) or atrial tachycardia at admission before treatment, age between 18 and 49 years, and a history of at least 3 SVT episodes. Exclusion criteria were defined as the presence of structural heart diseases such as heart failure, significant valvular disorders, hypertrophic cardiomyopathy, or myocardial infarction, as well as a history of malignant ventricular tachycardia or fibrillation. The study included 51 patients with active SVT (26 men, 25 women), all of whom were evaluated and sampled during the acute tachycardia episode prior to rhythm conversion, and a control group of 30 healthy individuals matched for age and sex (15 men, 15 women). Written informed consent was obtained from all individual participants included in the study.

### 2.2. Measurement of GPER, 8-iso-PGF2α, and tau protein levels

This study was conducted in collaboration between the Cardiology Outpatient Clinic and the Medical Biochemistry Research Laboratory of KSÜ Research Hospital. Venous blood samples were collected into EDTA-containing purple-top tubes from both the patient group diagnosed with SVT and the control group of healthy individuals. Venous blood samples from SVT patients were obtained immediately after electrocardiographic confirmation of active PSVT and prior to any pharmacologic or procedural intervention for arrhythmia termination. No post-conversion samples were collected. To obtain plasma, the blood samples were centrifuged at 4000 rpm for 15 minutes, and the supernatant was separated as plasma. GPER, Tau protein, and 8-iso-PGF_2_α levels in the obtained samples were measured using commercial ELISA kits (MyBioSource; MBS8802932, MBS022635, MBS263141). All analyses were performed according to the manufacturer’s protocol. Briefly, samples and standards were added to pre–antibody-coated microtiter plates, and following incubation, the plates were washed to remove unbound components. Subsequently, biotin-labeled secondary antibodies were added, followed by the addition of streptavidin-conjugated enzyme solution after a second incubation. After the final washing step, the substrate solution was added, and the resulting color change was allowed to develop at room temperature, with absorbance measured spectrophotometrically at 450 nm. Analyte concentrations were calculated in ng/mL and pg/mL using standard curves.

### 2.3. Statistical analysis

Statistical analyses were performed using GraphPad Prism (GraphPad Software, San Diego). Prior to comparative analyses, the distribution of all variables was assessed using the Shapiro–Wilk test to determine normality. For parameters that showed a normal distribution, differences between the 2 groups (arrhythmia patients and healthy controls) were evaluated using the Student *t* test. For variables that did not meet the assumption of normality, comparisons were performed using the Mann–Whitney *U* test. In addition, the diagnostic performance of 3 selected parameters was evaluated by receiver operating characteristic (ROC) analysis, and the area under the curve (AUC) values were calculated to assess their ability to discriminate between arrhythmia patients and control subjects. A *P*-value of < .05 was considered statistically significant.

Sample size adequacy was evaluated using G*Power software (version 3.1; Franz Faul, University of Kiel, Germany) according to the method described by Faul et al.^[13]^ The calculation was based on effect size estimates derived from a previously published study by Wu et al.^[14]^ which investigated oxidative stress biomarkers in patients with paroxysmal atrial fibrillation (53 patients) and healthy controls (30 subjects). Assuming a 2-tailed significance level of 0.05 and a target statistical power of 80%, the estimated minimum sample size was comparable to the number of participants included in the present study (51 patients with supraventricular tachycardia and 30 healthy controls), indicating that the study population was sufficient to detect statistically significant differences between groups.

## 3. Results

### 3.1. Clinical characteristics

In the present study, Pro-BNP levels were found to be higher in the SVT group compared to the control group (71.9 ± 34.2 vs 56.5 ± 31.9), and this difference was statistically significant (Table 1). In contrast, no statistically significant differences were observed between the groups in terms of Troponin T, CK-MB, TSH, and estradiol levels. Notable differences were observed with respect to oxidative stress and neurohormonal markers. 8-iso-PGF_2_α levels were significantly higher in the SVT group compared to the control group (34.1 ± 7.58 vs 9.50 ± 2.28; *P* < .001). Similarly, Tau protein (24.8 ± 5.67 vs 8.09 ± 4.71; *P* < .001) and GPER levels (5.33 ± 0.83 vs 2.72 ± 0.81; *P* < .001) were significantly increased in the SVT group. These findings indicate that Pro-BNP levels are elevated in patients with SVT, whereas no significant changes were observed in classical cardiac injury markers or thyroid function indicators. Furthermore, the results suggest that oxidative stress and GPER-related pathways may play a potential role in the pathophysiology of SVT.

**Table 1:** Comparison of biochemical parameters between the healthy control group and patients with supraventricular tachycardia (SVT).

	Control Mean ± SD	SVT Mean ± SD	*P-v*alue Control vs SVT
N	30	51	
Sex (M/F)	15/15	26/25	
Pro-BNP (pg/mL)	56.5 ± 31.9	71.9 ± 34.2	**.05**
Troponin T (ng/L)	7.36 ± 2.90	7.18 ± 3.87	.62
CK-MB (ng/mL)	3.03 ± 0.91	3.10 ± 0.81	.73
TSH (µIU/mL)	2.14 ± 0.89	1.99 ± 0.74	.58
Estradiol (pg/mL)	51.6 ± 26.3	58.5 ± 29.3	.28
8-iso-PGF_2_α (ng/mL)	9.50 ± 2.28	34.1 ± 7.58	**<.001**
Tau (pg/mL)	8.09 ± 4.71	24.8 ± 5.67	**<.001**
GPER-1 (ng/mL)	2.72 ± 0.81	5.33 ± 0.83	**<.001**

Statistically significant *P*-values are shown in bold (*P* < .05).

8-iso-PGF_2_α = 8-iso-prostaglandin F_2_ alpha, CK-MB = creatine kinase-MB isoenzyme, GPER = G protein-coupled estrogen receptor, Pro-BNP = N-terminal pro-B-type natriuretic peptide, SVT = supraventricular tachycardia, Tau = tau protein, Troponin T = cardiac troponin T, TSH = thyroid stimulating hormone.

### 3.2. *Plasma GPER, 8-iso-PGF*_*2*_*α, and tau levels*

**Figure 1.**
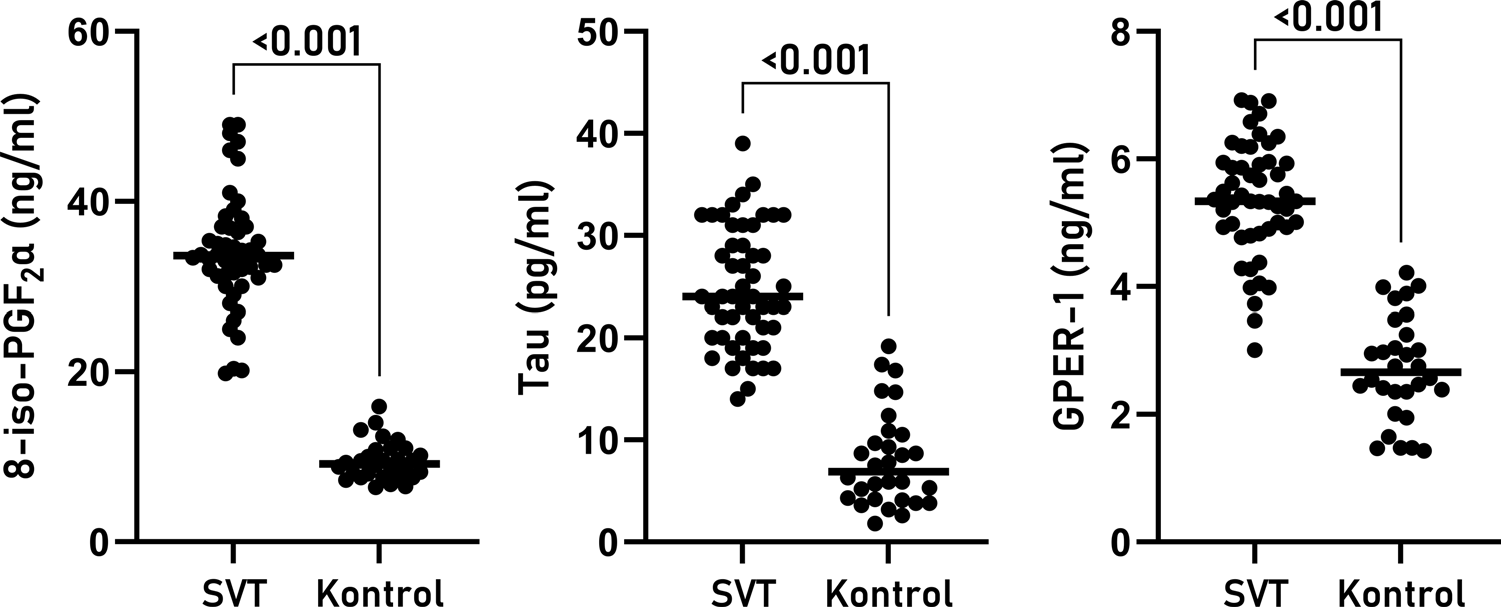
Plasma GPER, 8-iso-PGF_2_α, and tau levels in patients with supraventricular tachycardia. Plasma levels of 8-iso-PGF2α, Tau, and GPER were significantly elevated in the patient group compared with controls (***P < .001). 8-iso-PGF2α = 8-iso-prostaglandin F2 alpha, GPER = G protein-coupled estrogen receptor, SVT = supraventricular tachycardia, Tau = tau protein.

In our study, the levels of biomarkers related to oxidative stress, neuroinflammation, and cellular signaling were quantitatively evaluated by ELISA in patients with SVT. Levels of 8-iso-PGF_2_α, Tau, and GPER-1 were all significantly higher in the SVT group than in the control group (*P* < .001 for all biomarkers; Fig. 1). The significantly elevated levels of 8-iso-PGF_2_α in SVT cases indicate an increased oxidative stress response in these patients. The electrical load generated by rapid and recurrent atrial stimulation may lead to the accumulation of reactive oxygen species in cardiac myocytes. This process suggests that oxidative damage may play an active role in sustaining cardiac electrical instability.

Tau protein is a neuronal protein normally responsible for microtubule stabilization. However, its increase in systemic circulation is generally associated with neuroinflammation, neuronal injury, or cellular stress responses. The markedly elevated Tau levels in SVT patients indicate the effects of tachycardic episodes on the central nervous system and point to potential neurocardiac interactions. GPER is known to play a role in the cardiovascular system, particularly in vascular tone and cardiac remodeling processes. In our study, the significantly increased GPER levels in SVT patients suggest that this receptor may be involved in the tachycardic stress response and the pathophysiological mechanisms of SVT.

### 3.3. Diagnostic performance of biomarkers

**Figure 2.**
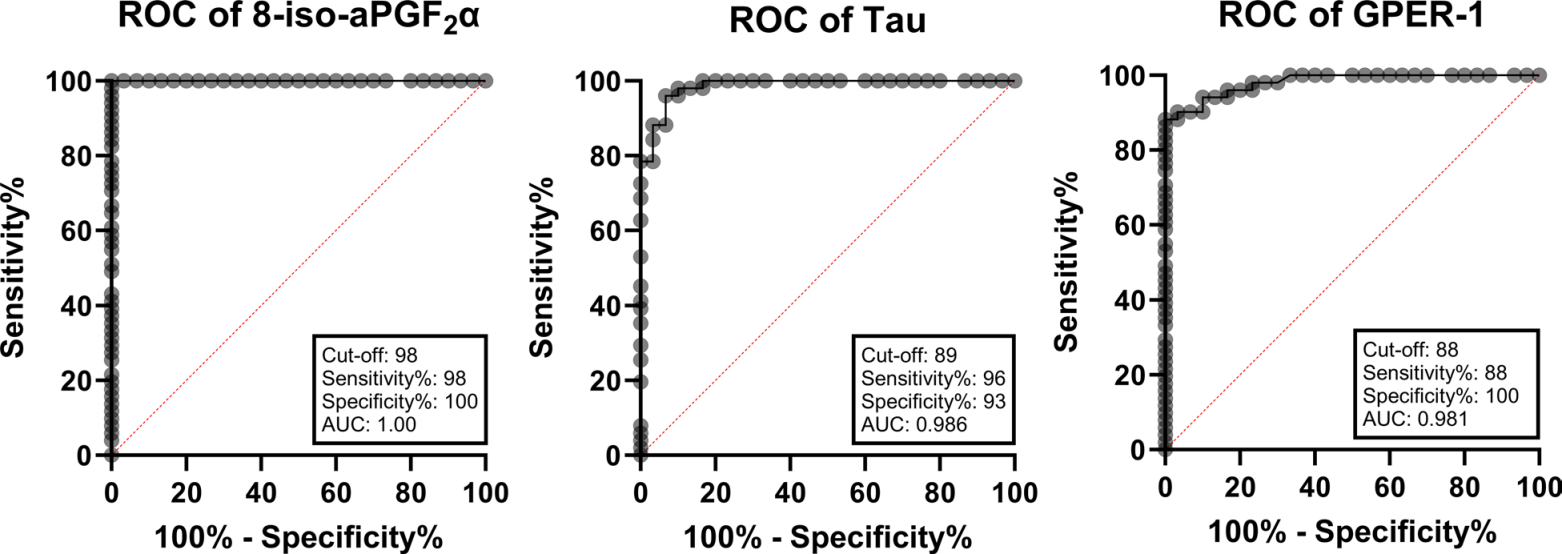
ROC curves of plasma GPER, 8-iso-PGF_2_α, and Tau in patients with supraventricular tachycardia. Cutoff values, sensitivity, and specificity for each biomarker are indicated in the graph. 8-iso-PGF2α = 8-iso-prostaglandin F2 alpha, AUC = area under the curve, GPER = G protein-coupled estrogen receptor, ROC = receiver operating characteristic Tau = tau protein.

ROC curve analysis was performed to evaluate the diagnostic value of 8-iso-PGF_2_α, Tau, and GPER biomarkers in SVT patients (Fig. 2). According to the ROC analysis, all 3 biomarkers demonstrated high discriminative performance. For 8-iso-PGF_2_α, a cutoff value of 98 ng/mL yielded 98% sensitivity, indicating strong diagnostic performance in identifying SVT cases (Table 2). For Tau protein, the AUC value was 0.986, with a cutoff value of 89 pg/mL yielding 96% sensitivity and 93% specificity. The diagnostic value of GPER levels was also high, with an AUC of 0.981. At a cutoff value of 88 ng/mL, sensitivity and specificity were 88% and 100%, respectively. These results indicate that 8-iso-PGF_2_α, Tau, and GPER levels emerge as potential biomarkers for SVT diagnosis, demonstrating high sensitivity and specificity.

**Table 2:** AUC values, optimal cutoff points, sensitivity, and specificity of 8-iso-PGF_2_α, Tau, and GPER-1 for predicting SVT.

Variables	[AUC] (CI: 95%)	Cutoff value	*P* value	Sensitivity (%)	Specificity (%)
8-iso-PGF_2_α	1.00	98	<.001	98	100
Tau	0.986	89	<.001	96	93
GPER-1	0.981	88	<.001	88	100

8-iso-PGF_2_α = 8-iso-prostaglandin F_2_ alpha, AUC = area under the curve, CI = confidence interval, GPER = G protein-coupled estrogen receptor, SVT = supraventricular tachycardia, Tau = tau protein.

## 4. Discussion

SVT is known as a type of arrhythmia originating from the atria or atrioventricular node, leading to rapid and irregular cardiac activity. Although it is generally associated with electrical abnormalities in the cardiac conduction system, the molecular mechanisms underlying these disturbances have attracted increasing attention in recent years. Research in this area has primarily focused on ion channel dysfunction, disturbances in calcium homeostasis, and vascular factors. In contrast, studies on oxidative stress, neuronal activity, and hormonal regulation axes remain quite limited. In this context, our study provides a comprehensive approach by focusing on GPER levels in SVT patients and simultaneously addressing biomarkers associated with oxidative stress and neurodegeneration.

In the pathophysiology of SVT, ROS play both a triggering and sustaining role. Oxidative species derived from mitochondrial and cytosolic sources target ion channels, calcium regulation, and intracellular signaling pathways, thereby creating a substrate for the formation of an arrhythmogenic microenvironment.^[15,16]^ In this context, ROS appear to function as active biological regulators shaping the molecular architecture of SVT. This view is supported by studies investigating nitric oxide (NO), nitric oxide synthase (NOS) isoenzymes,^[17]^ MDA–paraoxonase levels,^[18]^ and superoxide dismutase (SOD)^[19]^ in patient groups. In a study investigating the relationship between the NO-centered redox signaling network and atrial fibrillation, the pathological activation of iNOS was reported to create a proarrhythmogenic environment.^[17]^ In another study, elevated malondialdehyde (MDA) levels in patients with atrial fibrillation indicated increased lipid peroxidation, while high-density lipoprotein–associated paraoxonase (PON) levels reflected impaired antioxidant defense mechanisms.^[18]^ In a study examining the relationship between SOD deficiency and the pathogenesis of cardiac arrhythmia, decreases in SOD enzyme levels were observed across 8 different types of arrhythmias, including tachycardia, arrhythmia, and fibrillation, with the extent of reduction varying according to the arrhythmia type. In the SVT group, a 71% reduction in SOD levels was observed, indicating the presence of excessive oxygen radicals contributing to arrhythmogenesis.^[19]^ In a study investigating the relationship between atrial fibrillation and plasma levels of oxidative stress and inflammation markers, a positive correlation was observed between increases in patients’ left atrial diameter and pulmonary vein dimensions and ox-low-density lipoprotein and MDA levels, whereas SOD levels showed a negative correlation.^[20]^ Moreover, studies conducted in atrial fibrillation cohorts have reported that elevated 8-iso-PGF_2_α levels are associated with oxidative stress burden and adverse clinical outcomes such as thromboembolism.^[21–23]^ In a study examining 8-iso-PGF_2_α and NOX2 biomarker levels in individuals with atrial fibrillation, increases in both parameters were reported to be significantly associated with the development of cardiovascular complications and the risk of mortality.^[23]^ In another study evaluating the relationship between coagulation and fibrinolytic system markers and increased oxidative stress levels in atrial fibrillation, 8-isoprostane concentrations were reported to be significantly associated with thromboembolic events.^[21]^ Furthermore, in a study including 220 patients with paroxysmal and persistent atrial fibrillation, neutrophil/lymphocyte ratio, high-sensitivity hs-C-reactive protein, red cell distribution width, and 8-iso-PGF_2_α levels were shown to be significantly elevated in the patient groups. The findings of our study support the existing literature, and unlike previous studies, 8-iso-PGF_2_α, an oxidative stress biomarker, was measured directly for the first time in SVT patients, allowing a systematic assessment of the oxidative burden in this patient population.

Atrial fibrillation disrupts cerebral circulation and ischemic balance, leading to both damage in brain tissue and weakening of the blood–brain barrier. In this process, neuro-specific compounds enter the systemic circulation, reflecting the extent of neurological injury.^[24]^ Studies investigating the role of tau proteins in the brain–heart axis have been conducted in both human-derived samples and in vivo models.^[25–27]^ In a retrospective study involving 243 individuals, plasma levels of GFAP, S100B, GDF15, and Tau proteins – biomarkers of cerebral injury – were evaluated in subjects with and without atrial fibrillation. In patients with AF, significant increases in GFAP and Tau levels were observed. This increase has been reported to reflect the risk of cognitive impairment and the development of dementia in AF patients, in association with subclinical cerebral injury.^[25]^ In another study conducted on individuals with persistent atrial fibrillation, the relationship between cognitive dysfunction and both the duration of atrial fibrillation and anticoagulant therapy was investigated. The study indicated that elevated levels of T-tau, Aβ42, IL-6, and MMP-9 are associated with neurodegenerative and inflammatory processes underlying AF-related cognitive impairment.^[26]^ In a study using human myocardial and brain tissues obtained from individuals diagnosed with heart failure and Alzheimer disease, as well as a tauopathy mouse model, the distribution of tau proteins and the effects of monoclonal anti-tau antibody treatment were evaluated. Anti-tau antibody therapy was reported as a potential approach for the treatment of both myocardial and cerebral degenerative diseases.^[27]^ These studies demonstrate that tau protein is not only expressed in the brain in neurodegenerative diseases but also shows increased levels in diseased human cardiac tissues and in the systemic circulation following atrial fibrillation. Similarly, in our study on SVT cases, plasma Tau levels were found to be significantly elevated compared to healthy controls. This finding indicates that SVT extends beyond being merely a cardiac arrhythmia and may lead to biochemical changes associated with neuronal injury. Furthermore, our study is the first to demonstrate increased plasma Tau levels in SVT cases, a type of tachycardia that, unlike atrial fibrillation, does not carry a significant risk of mortality.

Through its role in mediating estrogen-related signaling pathways, GPER has been extensively investigated for its potential involvement in the pathophysiology and progression of various diseases.^[28,29]^ GPER is expressed in cardiomyocytes, vascular smooth muscle cells, and endothelial cells, and plays a critical role in regulating the physiological functions of the cardiovascular system.^[30]^ In the literature, selective activation of GPER via G1 and E2 has been shown to reduce L-type Ca^2+^ channel currents and cellular Ca^2+^ transients in male Wistar rat cardiomyocytes by activating the PI3K/AKT/NOS/NO pathway.^[8]^ These findings support the notion that GPER may exert a negative inotropic effect on cardiomyocyte contractility and potentially remodel the contraction mechanism at the cellular level.^[8]^ Similarly, in porcine cardiomyocytes, GPER activation via G1 has been reported to induce electrical changes in cardiac muscle cells and modulate calcium signaling in a manner that reduces arrhythmia risk. It has been suggested that, in the postmenopausal period, GPER may possess both regulatory effects on cardiac function and a protective potential against arrhythmias.^[31]^ In another study evaluating the clinical potential of estrogen-based therapeutic approaches against cardiac hypoxia, estrogen was reported to reduce cardiac complications via GPER activation.^[32]^ Although these studies reported in the literature provide findings based on experimental animal and cell models, there is no study directly investigating the relationship between GPER and human arrhythmias. In this regard, our study represents the first to evaluate GPER-related data in human SVT cases. The elevated GPER concentrations observed in SVT patients can be explained by 2 potential mechanisms. First, it may represent a compensatory protective response resulting from increased endogenous GPER expression in response to cellular stress and electrical imbalances occurring during arrhythmia. Second, it may reflect the release of GPER into the extracellular space due to disruption of cardiomyocyte membrane integrity associated with SVT; in this case, the elevated plasma levels could indicate myocyte injury.

## 5. Conclusion

This study is among the limited literature examining biomolecular changes associated with GPER, a key regulator of estrogen-mediated responses, in supraventricular tachycardia cases, together with markers of oxidative stress and neurodegeneration. When plasma GPER levels are evaluated together with the oxidative stress marker 8-iso-PGF_2_α and the neurodegeneration-associated protein Tau, they demonstrate strong diagnostic potential in distinguishing SVT patients from healthy controls. The fact that 8-iso-PGF_2_α, which is known to be associated with oxidative stress in atrial fibrillation, demonstrates a similar biomarker potential together with GPER in SVT supports the clinical relevance of this axis in supraventricular arrhythmias. The limited translational and clinical data on GPER and Tau protein in SVT and other supraventricular arrhythmias indicate that these molecules represent novel diagnostic targets warranting further investigation. Overall, GPER, 8-iso-PGF_2_α, and Tau emerge as biomarkers that may contribute to the more effective identification and stratification of patients experiencing frequent SVT episodes.

## 6. Limitations

One of the limitations of this study is that patients with SVT were evaluated as a single group, and different subtypes of SVT – such as atrioventricular nodal reentrant tachycardia (AVNRT), atrioventricular reentrant tachycardia (AVRT), and atrial tachycardia – were not analyzed separately. Since the underlying electrophysiological mechanisms of these arrhythmias may differ, the contributions of oxidative stress, neurodegenerative processes, and GPER-mediated signaling pathways may also vary by subtype. Therefore, the findings reflect general biomolecular changes associated with SVT. Future multicenter studies involving larger patient cohorts and detailed subgroup analyses may contribute to a better understanding of the roles of GPER, 8-iso-PGF_2_α, and Tau in different arrhythmia types, as well as the mechanisms associated with disease progression. Nevertheless, this study provides preliminary evidence supporting the association of these biomarkers with SVT and may guide future investigations.

## Author contributions

**Conceptualization:** Ünal Öztürk, Ergül Belge Kurutaş.

**Data curation:** Ünal Öztürk, Nuray Üremiş, Ergül Belge Kurutaş.

**Funding acquisition:** Ünal Öztürk.

**Investigation:** Nuray Üremiş, Ergül Belge Kurutaş.

**Methodology:** Ergül Belge Kurutaş.

**Project administration:** Ünal Öztürk.

**Resources:** Ünal Öztürk.

**Supervision:** Ünal Öztürk, Ergül Belge Kurutaş.

**Validation:** Ünal Öztürk.

**Visualization:** Nuray Üremiş.

**Writing – original draft:** Nuray Üremiş.

**Writing – review & editing:** Nuray Üremiş, Ergül Belge Kurutaş.
